# Photoinduced Rayleigh Light Scattering by Hyperbranched Poly(phenylene sulfide) Solutions: A Model for a Light Scattering Switch

**DOI:** 10.1021/acspolymersau.4c00044

**Published:** 2024-11-08

**Authors:** Usha Kalra, James E. Hanson

**Affiliations:** Department of Chemistry and Biochemistry, Seton Hall University, South Orange, New Jersey 07079, United States

**Keywords:** Photonics, Organic, Polymers, Scattering, Resonance Rayleigh, Fluorescence

## Abstract

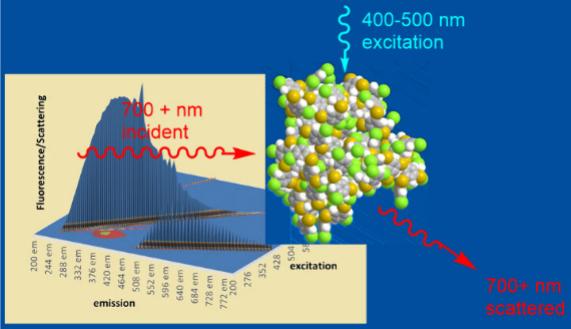

Unusual photophysical and scattering behavior is described for hyperbranched poly(phenylene sulfide) materials. These materials show interactions between the π systems of the aromatic monomer units in both ground and excited states, resulting in broad ranges for absorption and emission of light. The materials also display unusual scattering behavior; this is attributed to enhanced scattering by delocalized excited states resulting from the monomer unit interactions. This enhanced scattering is seen in the resonance Rayleigh scattering and second order scattering spectra. The ability to use these materials to construct a switch based on photoinduced scattering is demonstrated: application of ∼400 nm laser light increased scattering of 700 nm light by approximately 20%.

Materials with unique photophysical properties are required for the continuing development of photonics and optoelectronics.^1^ We report here the observation of unusual photophysical properties for hyperbranched poly(phenylene sulfide) materials, particularly photoinduced Rayleigh light scattering from organic materials in solution. These materials also show unusual absorption and emission photophysics, with a broad range of excitation wavelengths and emission wavelengths from multiple ground state and excited state interactions.

Hyperbranched poly(phenylene sulfide) (3,5-HPPS) was prepared from 3,5-dichlorobenzenethiol following a method we have previously reported^2^ and that has been subsequently employed by others as well.^3^ Two variations of the material were prepared: a standard material from polymerization of the monomer alone (3,5-HPPS) and a material prepared with 2-naphthalenethiol added at the end of the polymerization as an end-capping reagent (3,5-HPPS Np). Incorporation of extended aromatic structures such as naphthalene has been used by others to modify the photophysics of similar HPPS materials and other polymers.^3,4^ Structural descriptions and a summary of the characterization of these materials are provided in the Supporting Information, and full characterization is being submitted in a companion report.^5^

Absorbance spectra of the HPPS materials in dichloromethane (DCM) are shown in Figure 1. Observed absorbance extends out to almost 350 nm. Both polymers contain three types of benzene chromophores with either sulfur or chlorine substituents at the 1, 3, and 5 positions. These are denoted as terminal (“T”, 1 S and 2 Cl), linear (“L”, 2 S and 1 Cl), and dendritic (“D”, 3 S) following the conventions of hyperbranched polymers.^6^ HPPS Np also contains the 2-thionaphthyl end-cap groups (“N”). As individual molecules in dilute solution, these chromophores do not absorb at wavelengths longer than 300 nm (Supporting Information).^3,4,7^ We assign the longer wavelength absorbance observed in the polymers to direct excitation of ground state dimers or higher multimers that arise from π–π interactions between monomer units in the relatively compact structure of these polymers.^3−5,8^ These interactions are expected to be primarily within single polymer chains, not intermolecular, since the concentration used is very low (0.0035 g/L) and c* (the semidilute limit, where different polymer chains begin to touch in solution) for this molecular weight is calculated to be considerably higher at ∼180–330 g/L.^8,9^ These calculations may overestimate the value for c*, since there is evidence for concentration effects in the 1–10 g/L range.^3b^ Our samples are at least 3 orders of magnitude below c*. However, when the polymers are dissolved in good solvents such as DCM, the monomer units in the chains have the mobility to form π–π interactions intramolecularly within individual polymer chains. Although absorbance seems to disappear at wavelengths above 350 nm, there is a weak absorbance “tail” that continues to 400 nm or above, confirmed in the emission studies (*vide infra*).

**Figure 1:**
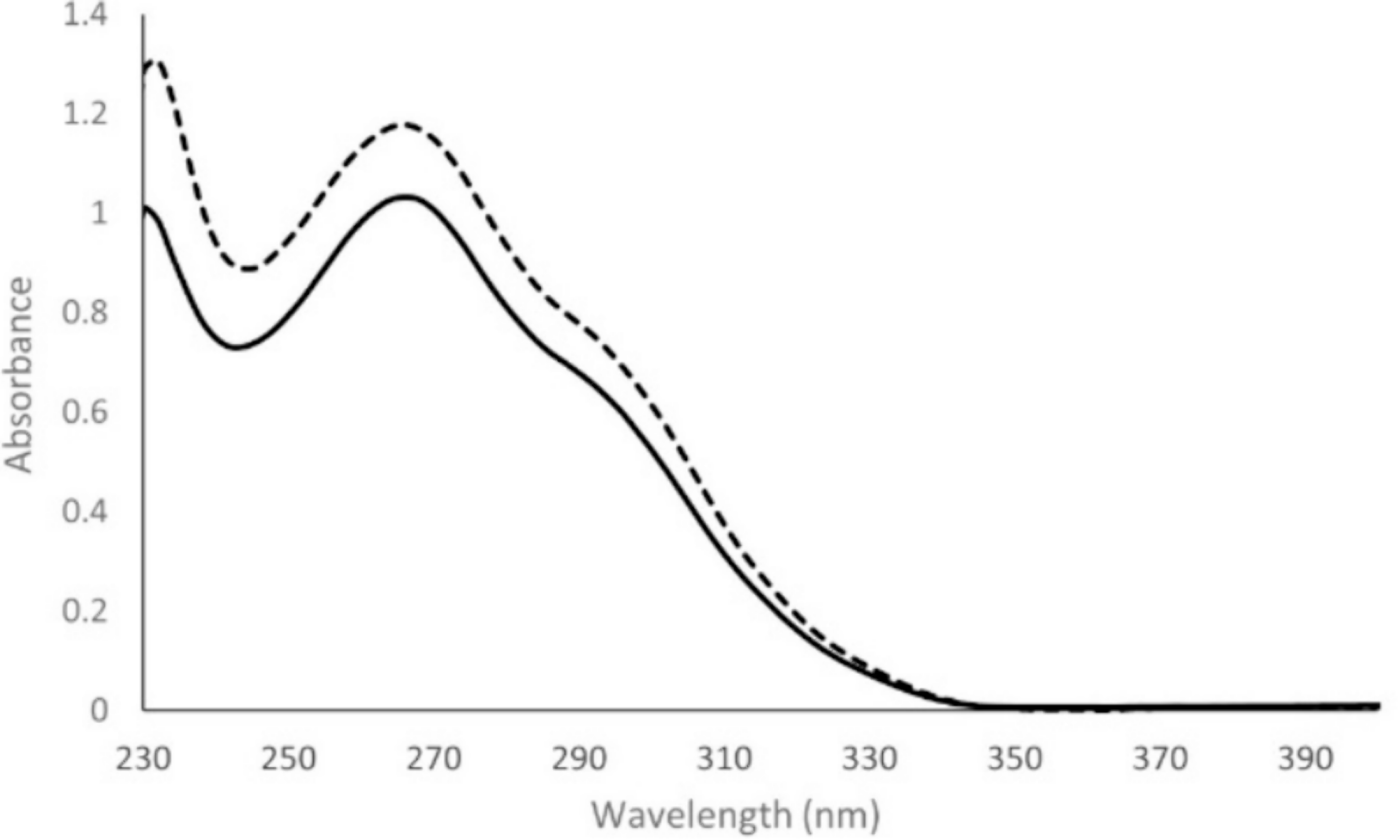
Electronic absorbance spectra of 3,5-HPPS (—), and 3,5-HPPS Np (- - -) in DCM, both spectra at 0.0035 g/L, ∼0.024 M concentration in chromophores (T, L, D, N).

Emission spectra are shown in Figure 2. When the excitation wavelength is low (250 to ∼300 nm), this excites the monomer units as individual aromatic chromophores, and emission is most intense in the wavelength range expected for the three types of aromatic chromophores (T, L, and D), with an emission maximum around 365 nm for 3,5-HPPS and 375 nm for 3,5-HPPS Np.^10^ The emission band is broader for 3,5-HPPS Np (80 nm at half height) than for 3,5-HPPS (70 nm at half height), not unexpected with a greater variety of chromophores found in 3,5-HPPS Np, including chromophores with larger π systems. The spectra for higher molecular weight 3,5-HPPS also show a second, lower maximum at about 450 nm. 3,5-HPPS Np shows emission at these longer wavelengths but does not show a clear maximum in this range when excited at 300 nm or below. As the excitation wavelength increases and the light is absorbed by ground state dimers and multimers, the emission maximum also shifts to longer wavelengths, close to this second maximum. When excited at 370 nm, the maxima for 3,5-HPPS and for 3,5-HPPS Np are both at approximately 440 nm. The emission from this longer wavelength excitation also extends to much longer wavelengths (past 600 nm). Note that this excitation (370 nm) is at longer wavelengths than the observed absorbance, and that the concentration is even lower than for the absorbance spectra (0.00018 g/L), which would preclude any intermolecular interactions: dimers and multimers are forming within polymer chains, not between them.

**Figure 2:**
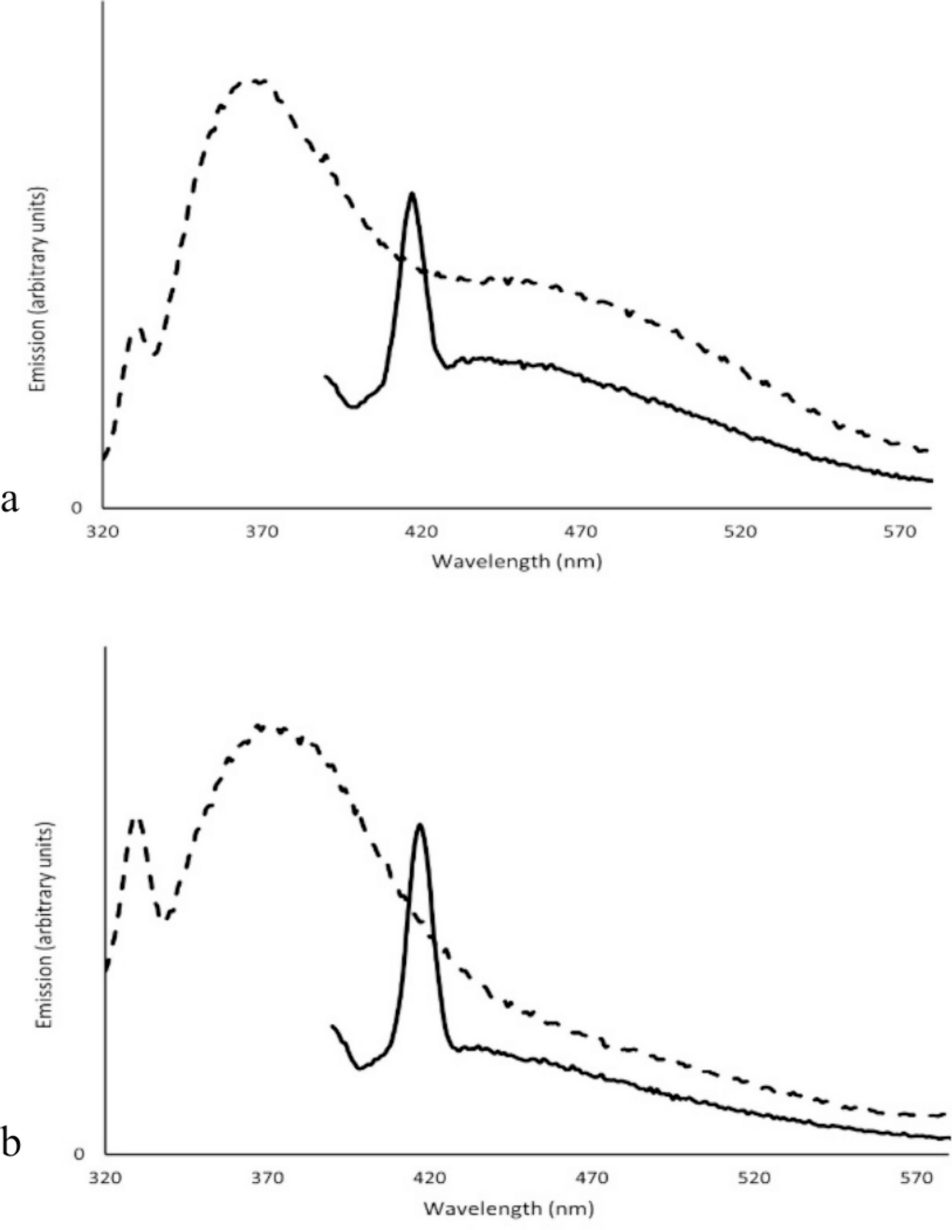
Emission spectra of (a) 3,5-HPPS and (b) 3,5-HPPS Np in CH_2_Cl_2_ (0.00018 g/L). Excitation at 300 nm (- - -) and 370 nm (—). The sharp peaks in parts (a) and (b) are Raman scattering from solvent (∼3000 cm^–1^ shift from excitation wavelength).

The photophysics of similar materials has been reported previously, but the earlier studies were not comprehensive. Yemul et al.^3a^ prepared PPS dendrimers and evaluated them by steady state fluorescence using relatively concentrated samples (1 mg/mL or 1 g/L) in dichloromethane, and exciting at 248 nm. They observed fluorescence maxima in the 375–390 nm range, similar to what we report here upon excitation at 300 nm. Xu et al.^3b^ prepared hyperbranched PPS materials and evaluated them in THF by a variety of fluorescence techniques, including steady state fluorescence and fluorescence lifetimes. They were excited at 370 nm, and fluorescence maxima were observed around 440 nm, again similar to what we observe when exciting at 370 nm. The concentration varied between 0.1 and 1 mg/mL (g/L), and some concentration dependence of fluorescence was observed, with λ_max_ shifting from about 435 to 465 nm. They attributed this to intermolecular excimer formation, but it is possible that these observations are from inner filter effects.^11^ We did not see changes in the emission maximum from 0.000002 to 0.2 g/L, as shown in the Supporting Information. Inner filter effects began around 0.2 g/L. Lifetimes in the range of 3–4 ns were obtained; as described in the Supporting Information, we observed multiple exponential decays that depend on detection wavelength, but included lifetimes in the 3–4 ns range. Note that our comprehensive measurements of fluorescence agree with BOTH of these reports: HPPS materials excited at lower wavelengths (220–325 nm) show fluorescence maxima in the 360–380 nm range, but the same materials excited at longer wavelengths where ground state dimers/oligomers/multimers absorb (350 nm and higher) give fluorescence maxima at 440 nm or longer. The observation of different emission spectra with different excitation wavelengths makes these materials good candidates for emission-excitation matrix spectra (as provided in the Supporting Information).

In acquiring Emission-Excitation Matrix (EEM) spectra to better understand the photophysics, it was observed that the HPPS solutions gave very intense Resonance Rayleigh (RR) scattering and Second Order (SO) scattering. These are shown in Figure 3, and the related EEM spectra are provided in the Supporting Information. The RR and SO data are provided as excess scattering above that observed for pure solvent. Measurement of RR scattering is extremely sensitive to any fluctuations in light intensity, and since these spectra are the difference in RR intensity of a sample relative to the RR intensity of pure solvent, fluctuations can occur in either measurement. The observed “noisy” data are therefore assigned to these random fluctuations, and we do not assign any significance to the occasional spikes in intensity. Strong RR scattering can be seen when light absorption couples with scattering, and can be correlated with certain effects of absorption: increased polarizability and increased molecular size.^12^ Significant RR scattering is seen for 3,5-HPPS at wavelengths up to and beyond 500 nm, well above the observed absorption range. We interpret this as evidence of weakly absorbing but strongly scattering ground state dimers and multimers. SO scattering can be an artifact of the emission monochromator but has more recently been studied as a nonlinear optical effect.^13,14^ The observation here that SO scattering has greatest intensity for excitation wavelengths between 300 and 400 nm (scattering observed from 600 to 800 nm) suggests that this may be a two-photon effect: scattering of a second photon from a molecule excited by the first photon.^14^ Excitation of the ground state dimers or multimers would generate a delocalized excited state that is expected to be more strongly scattering (larger scattering cross-section) than the ground state of the polymers or the excited states localized on a single monomer unit.^15^ This would correlate with the highly polarizable character of such a delocalized excited state.^12^ If even a small fraction of the sample exists in this strongly scattering excited state, then overall scattering could be greatly enhanced. Such a behavior would explain the observed RR and SO scattering data.

**Figure 3:**
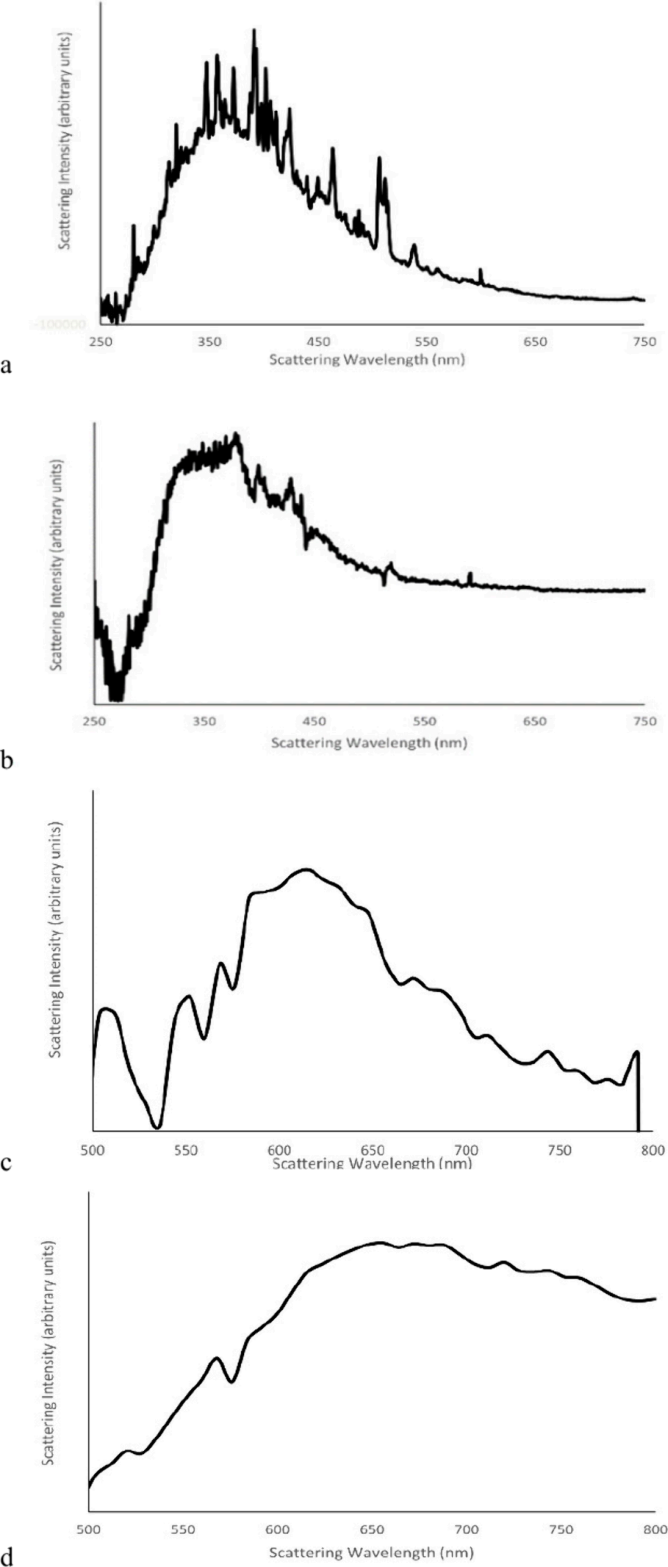
Resonance Rayleigh scattering of (a) 3,5-HPPS and (b) 3,5-HPPS Np in CH_2_Cl_2_ (0.00018 g/L). Second order scattering of (c) 3,5-HPPS and (d) 3,5-HPPS Np in CH_2_Cl_2_ (0.00018 g/L).

Photoinduced light scattering is a known phenomenon, first reported for inorganic solids.^16^ In optical fibers, this effect can lead to loss of intensity and degradation of the signal.^17^ There is a large body of literature on lithium niobate crystals, which show this behavior both as pure crystals and doped with other metal ions.^18^ Starting in the 1990s, photorefractive polymers were developed that showed this behavior as well.^19^ Most of this work has been performed in the solid state and with an applied static electric field, but our observations are in solution with no applied field.

This observed effect could be harnessed to make a photonic “switch” based on scattering. Scattering of longer wavelength light would be enhanced by excitation of the dimers and multimers with a shorter wavelength beam. To test this, we prepared a model of a photoinduced scattering “switch” using the fluorimeter. The excitation wavelength was set at 700 nm, and the emission was scanned from 695 to 705 nm to detect scattering of the excitation beam. As seen in Figure 3, the resonance Rayleigh scattering at 700 nm is very low—essentially zero. A 700 nm interference filter was placed in front of the emission monochromator to exclude all other wavelengths of light. A cuvette containing 0.0035 g/L 3,5-HPPS in dichloromethane was placed in the light path. A 405 nm blue laser was placed above the cuvette, aligned to direct the beam vertically down through the solution. Scattering was measured with the 405 nm laser off, then again with it on. Scattering increased by up to 20% when the 405 nm light was directed through the cuvette. The experimental setup and data are shown in Figure 4., and a photograph of the setup is provided in the Supporting Information. This switch can be understood in analogy to a triode device, where the 700 nm excitation is the emitter and the 700 nm scattered light is the collector, while the 405 nm input is the base. A similar observation was made for perovskite ceramics, where a maximum transient reduction of up to 80% in transmitted 632.8 nm light was observed when intense pulses of 532 nm light were applied. This reduced transmittance was assigned to increased scattering.^20^ It is not easy to compare this observation to ours, with a 20% increase in observed steady state scattering with application of a continuous wave laser.

**Figure 4:**
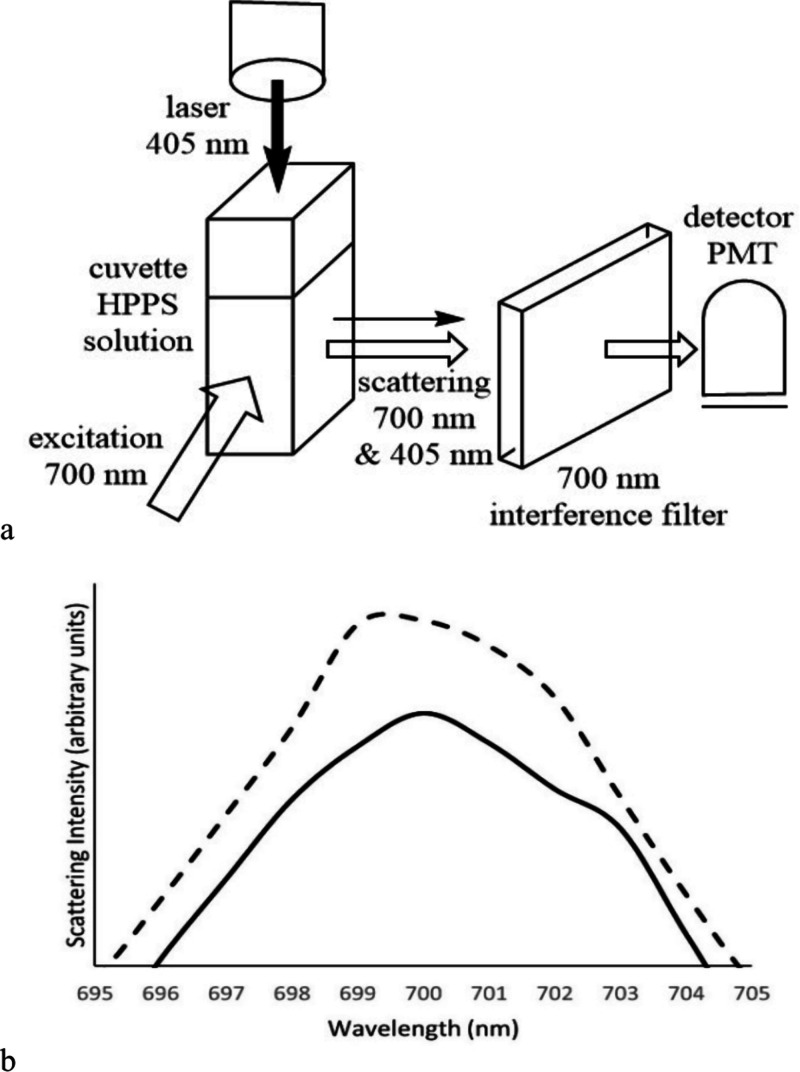
Model of a scattering based switch. (a) Diagram of the experimental apparatus using the fluorimeter. (b) Scattering intensity measured without 405 nm laser applied (—) and with 405 nm laser applied (- - -).

In conclusion, 3,5-branched hyperbranched polyphenylene sulfide materials were prepared and studied by optical techniques (absorbance, fluorescence, and scattering). The materials show unusual properties that we attribute to the formation of polarizable, delocalized excited states from ground state dimers and other multimers. These properties include unexpected long wavelength absorbance and fluorescence and strong RR and SO scattering. The observation of photoinduced scattering was used to prepare a model of a photonic switch based on this phenomenon. Such devices, modified to perform in the solid state, could be a useful addition to photonics technology.^21^ The unusual photophysics observed for these materials might also allow the development of other photonic devices.

## Supporting Information Available

Preparation and characterization of materials monomer absorbance spectra concentration dependence of emission spectra fluorescence lifetime data emission excitation matrix spectra image of light scattering switch mock-up (PDF)
