# Supplementary material for: Photoinduced Rayleigh Light Scattering by Hyperbranched Poly(phenylene sulfide) Solutions: A Model for a Light Scattering Switch
Source: ACS Polym Au. 2024 Nov 8;4(6):487–91. doi: 10.1021/acspolymersau.4c00044 (PMC11638783; doi:10.1021/acspolymersau.4c00044)
Supplement: Supplementary file 1 — lg4c00044_si_001.pdf [file lg4c00044_si_001.pdf]

Supporting Information

Photoinduced Rayleigh Light Scattering by Hyperbranched Poly(phenylene Sulfide) Solutions: A Model for a Light Scattering Switch

*Usha Kalra and James E. Hanson**

Department of Chemistry and Biochemistry, Seton Hall University, South Orange, NJ 07079 USA

James.Hanson@shu.edu

SUPPORTING INFORMATION

Preparation and Characterization of Materials page 2

Absorbance Spectra of Monomers page 5

Concentration Dependence of Emission Spectra page 6

Fluorescence Lifetime Data page 8

Emission-Excitation Spectra page 9

Image of Scattering Switch Mock-Up page 10

Preparation & Characterization of Materials

Except as noted, all chemicals and materials were purchased from commercial suppliers and used without further purification. SEC-LS analysis (including determination of [h] and Mark-Houwink parameters for calculating c*) was performed by PolyAnalytik Laboratory, London, Ontario, Canada. Elemental analysis was performed by Robertson Microanalytical, Ledgewood NJ. Infrared spectra were acquired on a Shimadzu IRSpirit using a QATR-S ATR cell with diamond prism. Electronic absorption (UV/vis) spectra were acquired on HEWLETT PACKARD 8452A diode array spectrophotometer. Electronic emission spectra were acquired on a HORIBA INSTRUMENTS FL-1000 Spectrofluorometer.

Standard Preparative Procedures for materials:

3,5-HPPS. 3,5-Dichlorobenzenethiol (1.00 g; 5.58 mmol) was added to anhydrous K_2CO_3 (4.63g; 33.5 mmol) in 10 mL of N-Methyl-2-pyrrolidone (NMP) in a 50 mL round bottom flask with magnetic stir bar. The reaction was purged with N_2, and the flask was heated to 150 °C in an oil bath in inert atmosphere (N_2 balloon) and maintained for the desired time (2 -12hr). The reaction was cooled, then diluted with 30 mL of ice water and 10 mL of 6 M HCl was then added to quench the reaction. The resulting precipitate was vigorously stirred for 1 h and then filtered. The precipitate was dried thoroughly under vacuum, then dissolved in a minimal amount (2-4 mL) of THF with occasional stirring using vortex. This THF solution was added dropwise to 80 mL of hexanes with vigorous stirring over a period of 2 hr. The resulting precipitate was then filtered, washed with hexanes, and dried thoroughly under vacuum. Isolated yields ranged from 0.213 g (27%, 2h) to 0.543 g (68%, 8 hr).

SEC-LS: M_n = 10.8 kD, M_w = 14.3 kD, M_w/M_n = 1.32; [η] = 0.0295 dL/g; R_g = 1.84 nm, Mark-Houwink: log K = -2.421, a = 0.215

DSC: T_g = 73.4 ºC

TGA: T_d (N_2) = 454.2 ºC

***3,5-HPPS Np*:** 3,4-Dichlorobenzenethiol (1 g; 5.58 mmol) and anhydrous K_2CO_3 (4.63g; 33.5 mmol) in 10 mL of NMP were combined in a nitrogen purged 50 mL round bottom flask. The reaction mixture was heated to 150 °C in an oil bath and this temperature was maintained for 6hr while in an inert atmosphere. After 6 hr, 2-Naphthalenethiol (0.89g; 5.56 mmol) was added to the reaction and the reaction was heated for an additional 2 hr at the same temperature while maintaining an inert atmosphere. The workup was the same as the general polymerization above. The product yield was 0.746 g (47%).

SEC-LS: M_n = 9.4 kD, M_w = 12.6 kD, M_w/M_n = 1.34; [η] = 0.0294 dL/g; R_g = 1.76 nm; Mark-Houwink: log K = -2.366, a = 0.201

DSC: T_g = 75.8 ºC

TGA: T_d (N_2) = 472.8 ºC

Fluorescence and Scattering Experiments.

Experiments were run at the concentrations noted. Emission spectra were run with 1 or 2 nm slits on both the excitation and emission monochromators (adjusted to keep the total counts below 10^6), and a 1 nm step. Data are presented in S/R mode to correct for lamp intensity. Resonance Rayleigh scattering was measured running the fluorimeter in Synchronous mode. Spectra are presented after subtracting the scattering from pure solvent (CH_2Cl_2). Second Order scattering was obtained from Emission-Excitation Matrix data with a 4 nm step for the grid and presented after subtracting

scattering from pure solvent (CH_2Cl_2). Other Emission-Excitation Matrix spectra were obtained using a 4 nm step for the grid. Fluorescence lifetimes were measured using single photon counting. The excitation wavelength was 350 nm. The emission monochromator was set at 400, 425, 475, 550 and 625 nm. Fluorescence decays could not be fit well to a single or double exponential model, but gave very good fits ($\chi^2 < 1.1$ in all cases) to a three exponential model. The three lifetimes were very short (0.1-0.7 nsec), short (1.25-3.75 nsec), and moderate (9-14 nsec) for all fits. The fraction assigned to a given lifetime was a fitted parameter, and shifted from mostly very short lifetimes at lower wavelengths to a slight excess of the short or moderate lifetime at longer wavelength.

Absorbance Spectra of Monomers

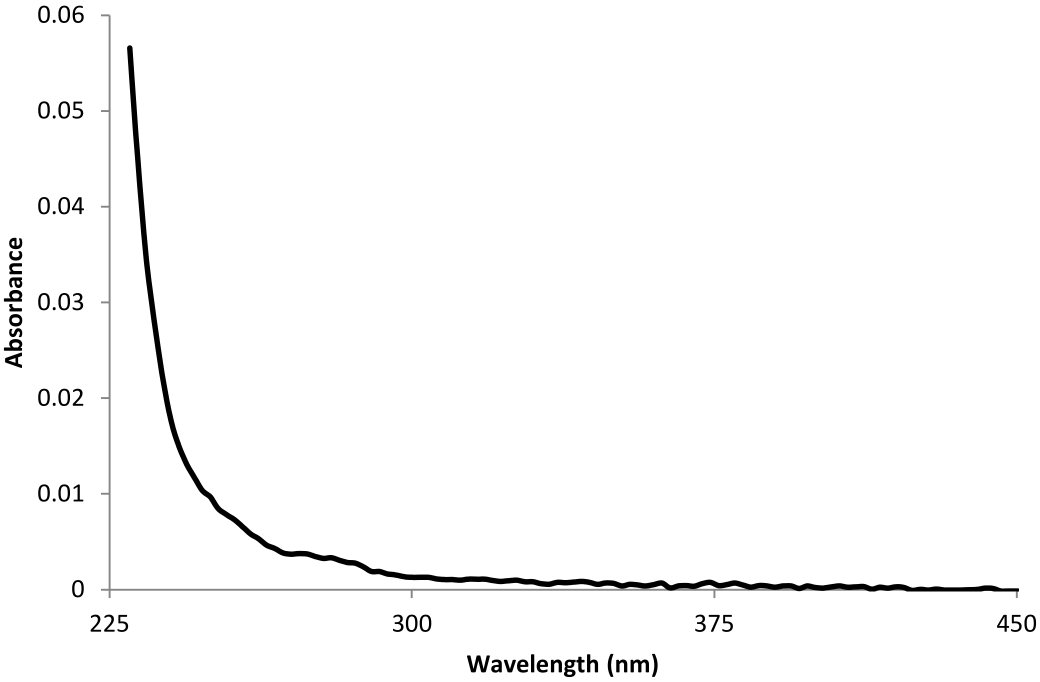

Figure S1. Absorbance spectrum for 3,5-dichlorothiophenol (monomer)

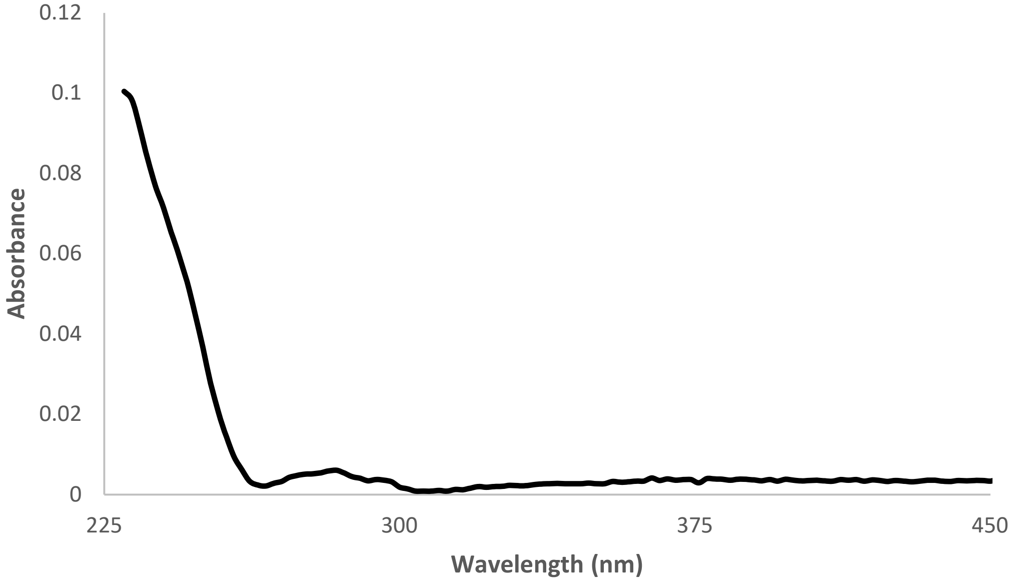

Figure S2. Absorbance spectrum for 2-naphthalenethiol (end cap)

Concentration Dependence of Emission Spectra

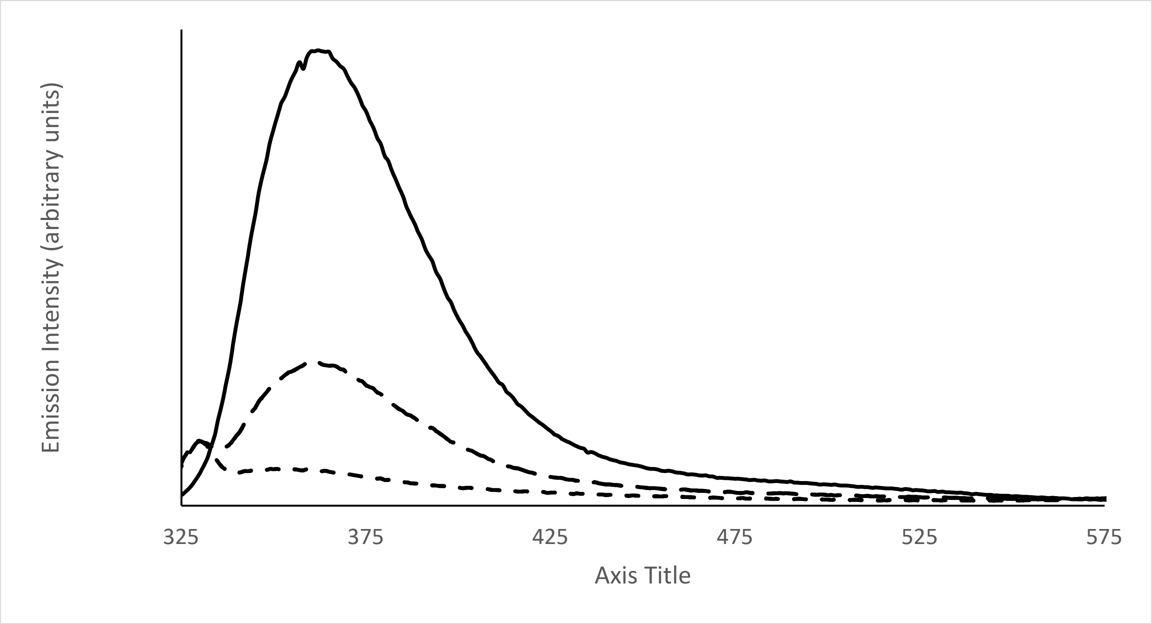

Figure S3. Emission spectra for **3,5-HPPS** excited at 300 nm: 0.02 g/L (——), 0.0002 g/L (– –) and 0.000002 g/L (- - -)

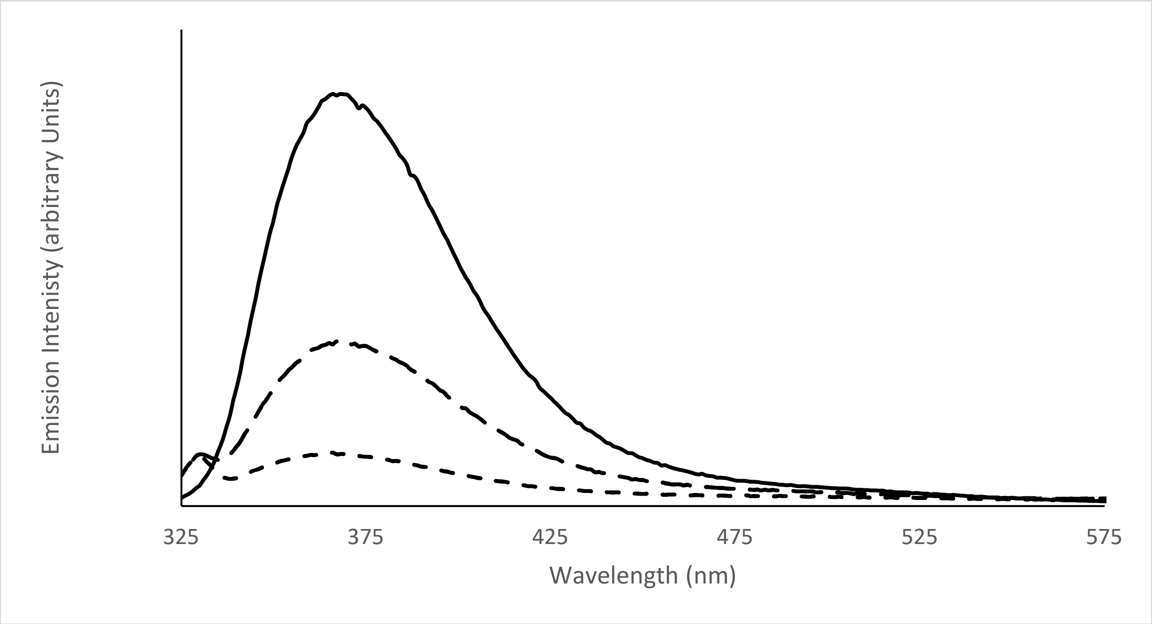

Figure S4. Emission spectra for **3,5-HPPS-Np** excited at 300 nm: 0.02 g/L (——), 0.0002 g/L (— —) and 0.000002 g/L (- · -)

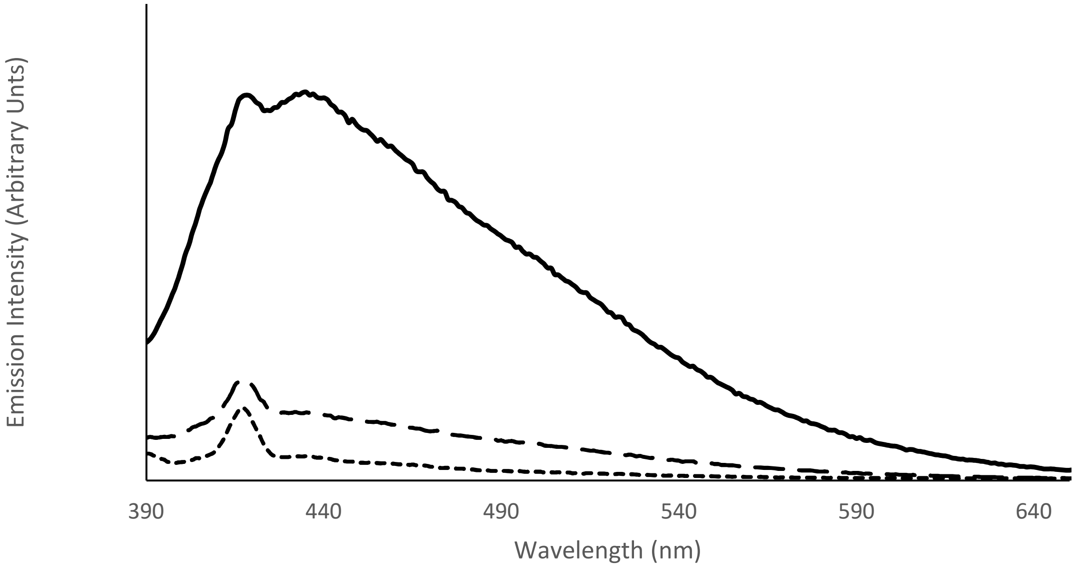

Figure S5. Emission spectra for **3,5-HPPS** excited at 370 nm: 0.2 g/L (——), 0.02 g/L (— —) and 0.0002 g/L (- - -)

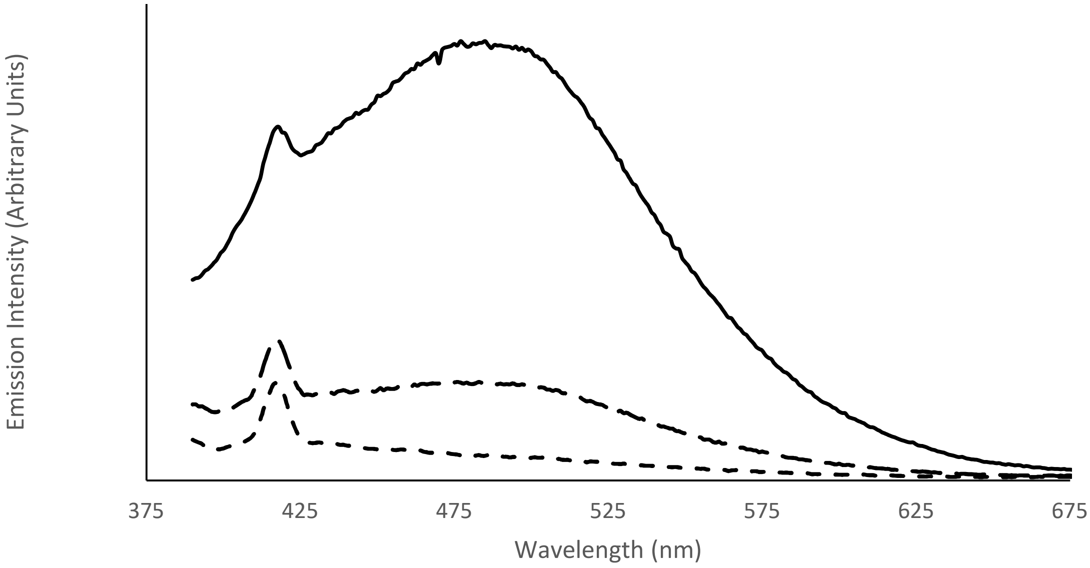

Figure S6. Emission spectra for **3,5-HPPS-Np** excited at 370 nm: 0.2 g/L (——), 0.02 g/L (— —) and 0.0002 g/L (- · - ·)

Fluorescence Lifetime Data

Table S1. Lifetimes (nsec) and fractions for triple exponential fits of emission decay in dichloromethane as a function of wavelength. Excitation 350 nm.

	λ (nm)	400	425	475	550	625
**3,5-HPPS**	τ1 (nsec)<br>(fraction)	0.18 +/- 0.01<br>(0.81)	0.27 +/- 0.01<br>(0.52)	0.49 +/- 0.01<br>(0.34)	0.46 +/- 0.01<br>(0.20)	0.16 +/- 0.01<br>(0.34)
	τ2 (nsec)<br>(fraction)	1.41 +/- 0.03<br>(0.17)	1.52 +/- 0.03<br>(0.39)	2.36 +/- 0.07<br>(0.40)	3.1 +/- 0.1<br>(0.38)	2.4 +/- 0.1<br>(0.29)
	τ3 (nsec)<br>(fraction)	11.7 +/- 0.5<br>(0.02)	9.1 +/- 0.1<br>(0.09)	11.0 +/- 0.1<br>(0.26)	11.7 +/- 0.1<br>(0.42)	10.6 +/- 0.2<br>(0.37)
**3,5-HPPS Np**	τ1 (nsec)<br>(fraction)	0.23 +/- 0.01<br>(0.84)	0.26 +/- 0.01<br>(0.71)	0.51 +/- 0.01<br>(0.32)	0.63 +/- 0.01<br>(0.15)	0.21 +/- 0.02<br>(0.22)
	τ2 (nsec)<br>(fraction)	1.28 +/- 0.03<br>(0.14)	1.50 +/- 0.04<br>(0.24)	3.15 +/- 0.05<br>(0.45)	3.75 +/- 0.07<br>(0.50)	2.70 +/- 0.06<br>(0.37)
	τ3 (nsec)<br>(fraction)	9.3 +/- 0.4<br>(0.02)	9.3 +/- 0.2<br>(0.05)	13.9 +/- 0.1<br>(0.24)	13.1 +/- 0.1<br>(0.35)	10.7 +/- 0.1<br>(0.41)

Emission Excitation Matrix spectra

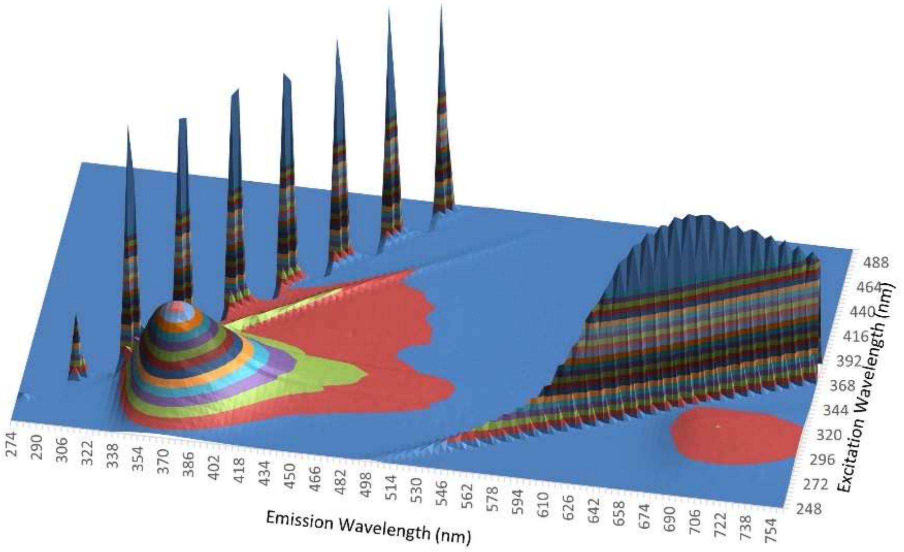

a)

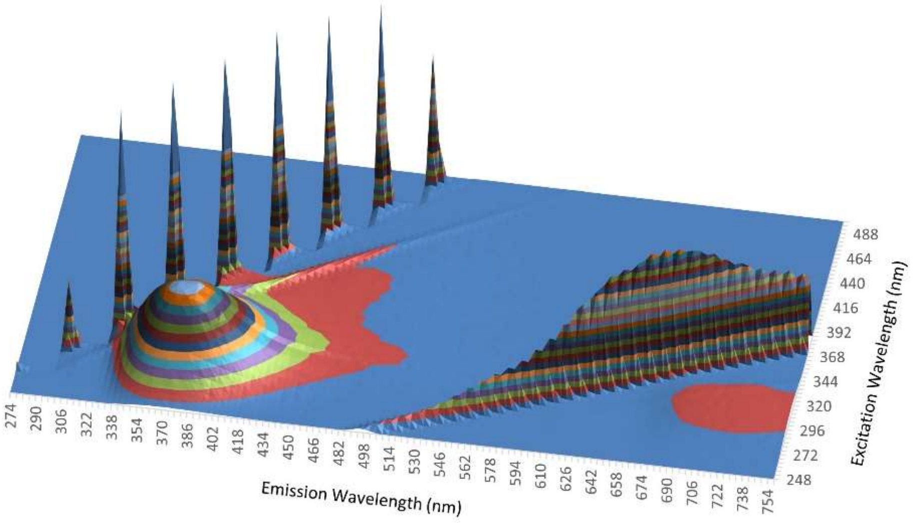

b)

Figure S7. Emission-Excitation Matrix spectra for (a) **3,5-HPPS** in dichloromethane, (b) **3,5-HPPS Np** in dichloromethane. Polymers are 0.0035 mg/mL and contour lines are at the same intensity levels in all spectra. The intense peaks along the diagonal are the edge of the Resonance Rayleigh scattering, while the intense line of scattering in the lower right corner is second order scattering. Note that excitation wavelengths are active to over 400 nm and emission tails out to well over 500 nm.

Image of switch mockup

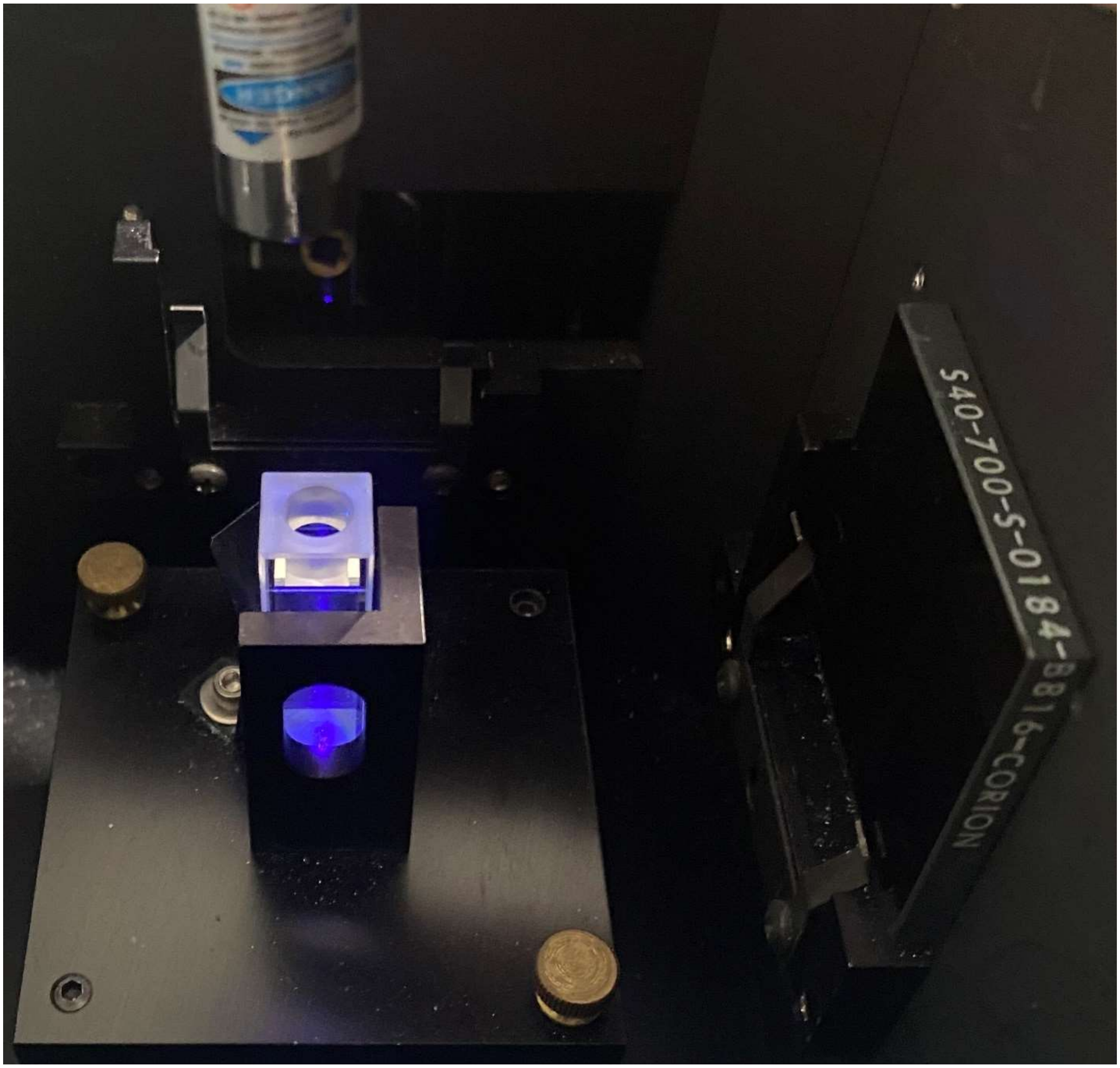

Figure S8. Photograph of switch mockup using fluorimeter. Cuvette, vertical laser, and interference filter are all visible. The 700 nm light enters from the front and is scattered at right angles, passing through the interference filter. The ~400 nm blue laser light is excluded by the interference filter.
